# A novel multi-component protein vaccine ECP001 containing a protein polypeptide antigen nPstS1 riching in T-cell epitopes showed good immunogenicity and protection in mice

**DOI:** 10.3389/fimmu.2023.1138818

**Published:** 2023-04-21

**Authors:** Jinjie Yu, Xueting Fan, Xiuli Luan, Ruihuan Wang, Bin Cao, Chengyu Qian, Guilian Li, Machao Li, Xiuqin Zhao, Haican Liu, Kanglin Wan, Xiuqin Yuan

**Affiliations:** ^1^ School of Public Health, University of South China, Hengyang, China; ^2^ State Key Laboratory for Infectious Disease Prevention and Control, National Institute for Communicable Disease Control and Prevention, Chinese Center for Disease Control and Prevention, Beijing, China; ^3^ School of Life Sciences, College of Laboratory Medicine, Wenzhou Medical University, Wenzhou, China

**Keywords:** mycobacterium tuberculosis, ESAT-6, CFP-10, nPstS1, adjuvant, recombinant subunit protein vaccine

## Abstract

Tuberculosis (TB) is an infectious disease that seriously affects human health. Until now, the only anti-TB vaccine approved for use is the live attenuated *Mycobacterium bovis (M. bovis)* vaccine — BCG vaccine, but its protective efficacy is relatively low and does not provide satisfactory protection against TB in adults. Therefore, there is an urgent need for more effective vaccines to reduce the global TB epidemic. In this study, ESAT-6, CFP-10, two antigens full-length and the T-cell epitope polypeptide antigen of PstS1, named nPstS1, were selected to form one multi-component protein antigens, named ECP001, which include two types, one is a mixed protein antigen named ECP001m, the other is a fusion expression protein antigen named ECP001f, as candidates for protein subunit vaccines. were prepared by constructing one novel subunit vaccine by mixing or fusing the three proteins and combining them with aluminum hydroxide adjuvant, and the immunogenicity and protective properties of the vaccine was evaluated in mice. The results showed that ECP001 stimulated mice to produce high titre levels of IgG, IgG1 and IgG2a antibodies; meanwhile, high levels of IFN-γ and a broad range of specific cytokines were secreted by mouse splenocytes; in addition, ECP001 inhibited the proliferation of Mycobacterium tuberculosis in vitro with a capacity comparable to that of BCG. It can be concluded that ECP001 is a novel effective multicomponent subunit vaccine candidate with potential as BCG Initial Immunisation-ECP001 Booster Immunisation or therapeutic vaccine for M. tuberculosis infection.

## Introduction

Tuberculosis (TB) is an infectious disease caused by *Mycobacterium tuberculosis* (Mtb) infection, which is one of the leading causes of death among infectious diseases currently (1–3). 10.6 million new cases of TB were reported globally in 2021, with an incidence rate of 134 per 100,000, of which 6.6% were co-infected with HIV, and an estimated 1.6 million deaths from TB worldwide in 2021 (3). In March 1993, the World Health Organization (WHO) declared tuberculosis to be a “global public health emergency” (4). In recent years, with the emergence of multidrug-resistant tuberculosis (MDR-TB) and the prevalence of HIV-merged TB infection, the prevention and control of tuberculosis has faced a more serious challenge (5, 6). Vaccination is the most effective strategy to prevent and control infectious diseases. The only TB vaccine currently licensed for use worldwide is the bacillus Calmette-Guerin (BCG) vaccine, a live attenuated vaccine for *Mycobacterium bovis* (*M. bovis*), which was first used in 1921 and is the most widely used vaccine in history worldwide (7). BCG vaccine is effective in preventing severe tuberculosis in children, such as miliary tuberculosis and tuberculous meningitis (8). However, the BCG vaccine showed significantly different levels of protection between populations and regions, with efficacy ranging from 0% to 80% in adults (9). In addition, the BCG vaccine, as a live vaccine, is potentially virulent and cannot be used in all populations (10). Therefore, there is an urgent need to develop new and more effective TB vaccines and immunization strategies to protect people from Mtb infection and TB treatment for promoting TB control worldwide.

In recent years, with the rapid development of molecular biology and vaccinology, several new TB vaccines have emerged, including inactivated, live recombinant, live attenuated, subunit protein and DNA vaccines. Among them, subunit vaccines have the advantages of high safety, good stability and low toxicity, and are one of the promising vaccination strategy (11). Currently, there have at least been 16 TB vaccine candidates in Phase I-III clinical trials worldwide, in which there are five protein subunit vaccines, including M72 (*Rv1196 + Rv0125*), H1 (*Rv1886 + Rv3875*), H4 (*Rv1886 + Rv0288*), ID93 (*Rv2608 + Rv3619 + Rv3620 + Rv1813*) and H56 (*Rv1886 + Rv3875 + Rv2660*). Studies have shown that TB subunit vaccines consisting of multiple protein mixtures, recombinant fusion proteins, or epitope tandem proteins of dominant antigens can induce stronger CD4^+^ T cell responses and greater protective efficacy than single protein subunit vaccines (12–14).

Early secretory antigentic target 6 (ESAT-6), coded by *Rv3875*, is an early secreted low molecular weight protein of Mtb with strong cellular immunogenicity, which plays an important role in the immune response against Mtb infection and is one of the main antigens recognized by the body’s immune system. ESAT-6 protein can induce strong T-cell responses, such as causing the body to secrete high levels of gamma-interferon (IFN-γ), which effectively activates macrophages to control Mtb infection (15, 16). The 10 kD culture filtrate protein (CFP-10), coded by *Rv3874*, is also a dominant antigen protein secreted early by Mtb. It has been shown that CFP-10 is a potent CD8^+^ T-cell antigen and significantly stimulates T cells to secrete IFN-γ (17). As major immunodominant antigens, ESAT6- and CFP-10 have 100% coverage of T-cell epitopes. Both are present in the differential region 1 (RD1) of Mtb and *M. bovis* virulent strains and are absent in BCG (18). Studies have shown that the lack of RD1 region is the main reason for the lack of BCG virulence (19). Currently, the design of TB vaccines and diagnostic reagents based on these two proteins has become a hot spot and a major direction in TB research (20). A periplasmic phosphate-binding lipoprotein PstS1 (phosphate transport subunit S1), coded by *Rv0934*, is a membrane transporter protein with a relative molecular mass of 38×10 (3) and is a phosphate-transporting lipoprotein encoded by the Mtb phosphate-transporting lipoprotein membrane-associated complex gene, which contains specific T and B lymphocyte antigenic epitopes and induces strong cellular and humoral immune responses in the body (21–23). In addition, Although PstS1 is the virulence factor of Mtb, it is a dominant antigen protein, and has good immunogenicity and immune protection, so it can protect mice from Mtb infection (24–26). As a component of protein antigens, antigenic epitopes are the core of protein antigenicity and play a decisive role in the process of antigen-induced immune response in the body. Antigens bind to the corresponding lymphocyte surface receptors in the body through antigenic epitopes to sensitize lymphocytes and induce an immune response in the body (27). The construction of antigenic epitope proteins allows the aggregation of immunodominant epitopes, shortens the length of antigens, removes redundant sequences from antigens, improves the immunogenicity of vaccines, and reduces side effects (28). Antigenic epitope proteins of Mtb are important for the development of subunit prophylactic vaccines. The T-cell epitope protein nPstS1 of PstS1 was constructed in our laboratory based on the predicted T-cell epitope of PstS1 combined with the immunogenicity assessment of the epitope peptide and the distribution of the epitope. NPstS1 was experimentally validated to have good immunoreactivity and to be more immunodominant than PstS1, making it suitable as a candidate antigen for TB vaccines (29). However, subunit vaccines consisting mainly of proteins or peptides may face limitations in terms of immunogenicity. Therefore, they require immunostimulatory adjuvants or delivery systems to enhance the immune response (30). The aluminum adjuvant is the only FDA-approved adjuvant for human vaccines (31). It strongly adsorbs protein antigens from the solution and forms a precipitate. When inoculated into the body, it forms an “antigen reservoir” that slowly releases the antigen, fully prolonging its duration of action. It also promotes the response of local (injection site) macrophages. Whether aluminum adjuvants can stimulate T-cell responses is unclear. Traditionally, it is widely believed that aluminum adjuvants trigger Th2-type immune responses. However, recent findings have shown that aluminum adjuvants can enhance Th1 and Th2 cell responses to antigens through the appropriate vaccination route (32, 33).

In this study, ESAT-6, CFP-10, two antigens full-length and the T-cell epitope polypeptide antigen of PstS1, named nPstS1, were selected to form one multi-component protein antigen, named ECP001, which include two types, one is a mixed protein antigen named ECP001m, the other is a fusion expression protein antigen named ECP001f, as candidates for protein subunit vaccines. We expressed and purified ESAT-6, CFP-10 and nPstS1, respectively, and made a multi-antigen mixture ECP001m by mixing these three proteins in equal proportions, and a fusion protein ECP001f by genetic engineering techniques. Both ECP001m and ECP001f were used as antigens, formulated with alum adjuvant, respectively, of which the immunogenicity and efficacy were analyzed by animal immune experiments with BALB/c mice, and the protective effects were evaluated using *Mycobacterium tuberculosis in vitro* growth inhibition assay (MGIA).

## Materials and methods

### Ethics

In this study, all animal experiments were conducted under the guidelines of the China Animal Protection Commission and were approved by the Animal Welfare Ethics Committee of Chinese Center for Disease Control and Prevention (No.2019-CCDC-IACUC-011).

### Experimental design and procedure

**Figure 1:**
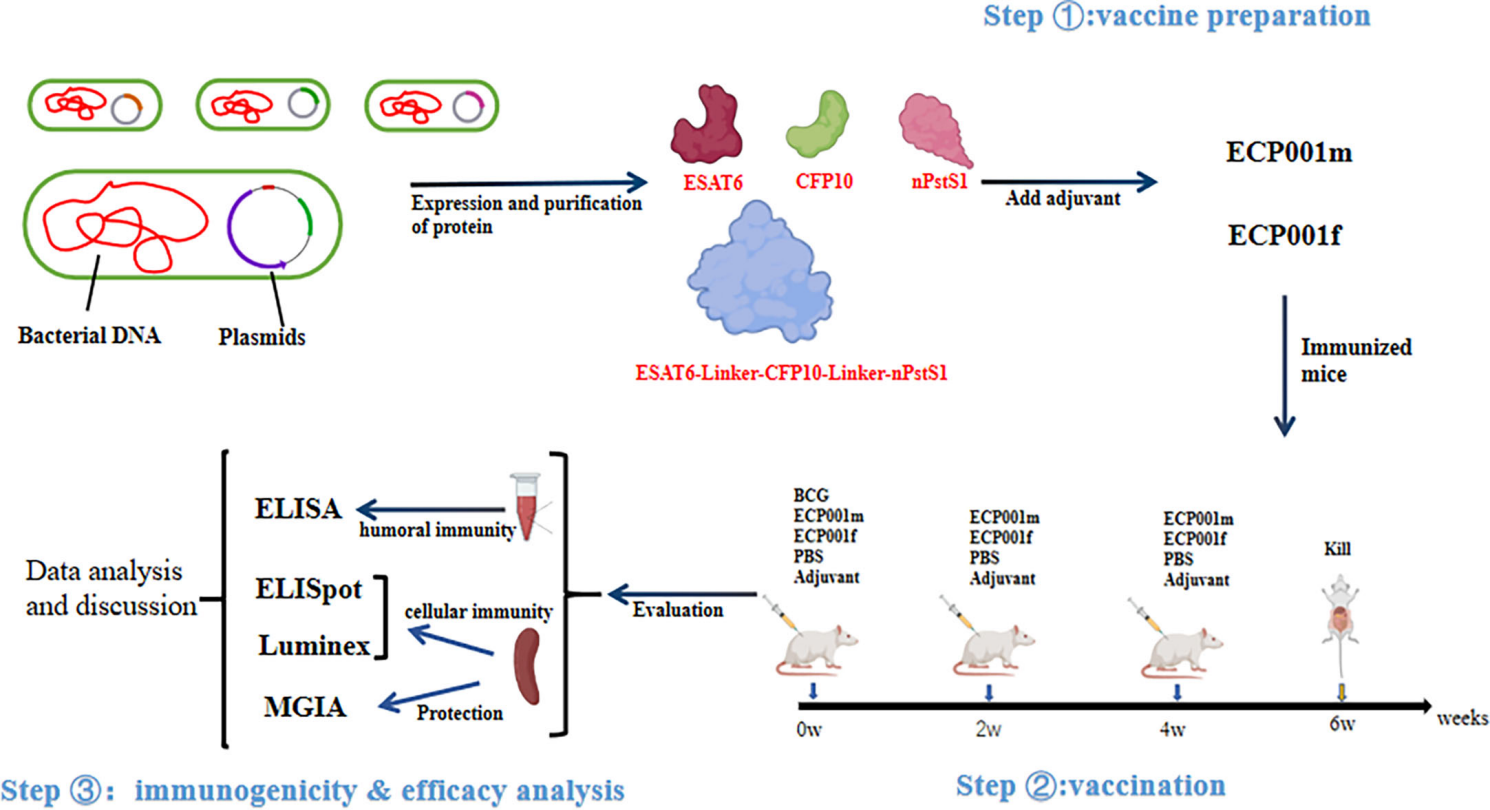
Schematic diagram of experimental design and procedures in this study.

**Figure 1** shows the experimental design and procedure of this study, which mainly includes three aspects, vaccine preparation, vaccination and immunogenicity & efficacy analysis.

### Experimental animal

30 healthy female BALB/c mice aged 6-8 weeks, were purchased from Beijing-based HFK Biotech Co. The mice were kept in pathogen-free, temperature-appropriate conditions at Laboratory Animal Center, Chinese Center for Disease Control and Prevention.

### Prediction of T-cell epitopes and selection of T-cell epitope-rich sequences

**Figure 2:**
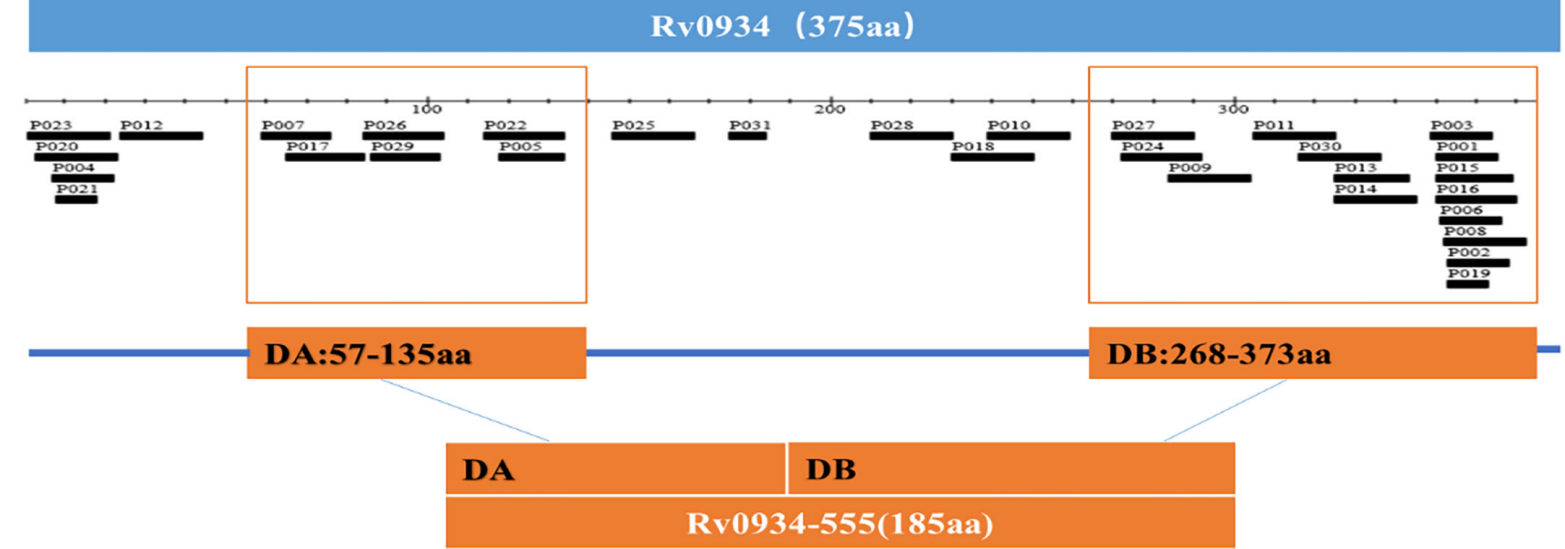
T-Cell epitope-rich domains distributed in *Rv0934*. The scale unit is amino acid (aa).

The *Rv0934* gene sequence of Mtb was obtained from the National Center for Biotechnology Information (NCBI) at https://www.ncbi.nlm.nih.gov The T cell epitope prediction software TEpredict and IEDB-AR (http://tools.iedb.org/mhci/) were used to predict T cell epitopes in *Rv0934* that can bind to human HLA-A02 supertype alleles (including HLA-A*0201, *0202, *0203, *0206 motifs). The immunogenicity of the epitopes was evaluated using the T-cell pMHC-I class immunogenicity predictor (http://tools.immuneepitope.org/immunogenicity/). In this study, we selected two concentrated regions enriched in *Rv0934* T-cell epitopes. The nucleotide positions of concentration region A are positions 169-405, and the nucleotide positions of concentration region B are positions 802-1119. The two fragments were spliced together to obtain a new gene DNA sequence of 555 bp in length, which was named *nRv0934*, and the corresponding protein was named nPstS1. The nucleotide and amino acid positions of *nRv0934* are shown in **Table 1**, and the two predicted T cell epitope concentration regions contained in *nRv0934* are shown in **Figure 2**.

**Table 1:** Nucleotide and Amino acid positions of the selected Rv0934-555 enriched with T-cell epitopes.

	Start1 at	End1 at	Length1	Start2 at	End2 at	Length2	Total length
Nucleotide sequence	169	405	237	802	1119	318	555bp
Amino acid sequence	57	135	79	268	373	106	185aa

### Strain and vector

Mtb H37Rv (ATCC 27294) and BCG-China (D2PB302) were provided by Tuberculosis Laboratory of National Institute of Communicable Disease Control and Prevention, Chinese Center for Disease Control and Prevention. Escherichia coli (*E.coli*) BL21 (DE3) and *E.coli* DH5αcompetent cells were purchased from Beijing Quanshijin Biotechnology Co., Ltd and Beijing Tiangen biochemical Technology Co., Ltd, Respectively. pET32a and pET43.1a plasmids were purchased from by Beijing Aoko Biotechnology Co. Ltd.

### Gene amplification, expression and purification of single proteins ESAT-6, CFP-10, nPstS1 and fusion protein ECP001f

The plasmids for the individual proteins in this study were constructed as follows: the DNA sequences of *Rv3875* and *Rv3874* genes were amplified by polymerase chain reaction (PCR) using H37Rv genomic DNA as the template, and the DNA sequences of *the nRv0934* gene was synthesized by Beijing Aoko Biotechnology Co. Ltd. The two ends of the primers included two enzyme cleavage sites, *EcoRI* and *HindIII*, and the list of primers is shown in **Table 2**. After double digestion by *EcoRⅠ* and *HindIII*, the target genes *Rv3875* and *Rv3874* were ligated into the expression vector pET32a, respectively. The plasmid of the fusion protein ECP001f was constructed as follows: the ECP001f plasmid consisted of three sequentially linked in tandem genes, *Rv3875*, *Rv3874* and *nRv0934*. To ensure that the spatial structure of each protein does not affect each other, a linker with the sequence “GGSGG” was added between each gene, and the outermost gene was connected with *NdeⅠ* and *XhoⅠ* enzymes cutting sites at both ends respectively. The genes were ligated and then cloned into vector pET43.1a.

**Table 2:** The primer sequence table of ESAT-6、CFP-10 and nPstS1.

Protein/Gene	Primer sequences	Enzyme cut site
CFP-10/*Rv3875*	F:5’-TACCG **GAATTC** ATGGCAGAGATGAAGACCGA-3	*EcoRⅠ*
	R:5’-ATCCC **AAGCTT** TCAGAAGCCCATTTGCGAG-3’	*HindIII*
ESAT-6/*Rv3874*	F:5’-TAT **GAATTC** ATGACAGAGCAGCAGT-3’	*EcoRⅠ*
	R:5’-ATCCC **AAGCTT** TGCGAACATCCCAGTGAC-3’	*HindIII*
nPstS1*/nRv0934*	F1:5’-CCG **GAATTC** GGTAGCACGCTGCTCTA-3’	*EcoRⅠ*
	R1:5’-ATCTCCGCTCAGCAGGTCAACTACAACAATAGCTCTGGCAATTTCTTGTT-3’	
	F2:5’-AACAAGAAATTGCCAGAGCTATTGTTGTAGTTGACCTGCTGAGCGGAGAT-3’	
	R2:5’-CCC **AAGCTT** GGAAATCGTCGCGATCA-3’	*HindIII*

“_____“ is the enzyme cut site.

All constructed recombinant plasmids were first transferred into *E. coli* DH5α receptor cells for amplification, and after verification by PCR, the recombinant plasmids were extracted and the concentrations were measured, and then the extracted recombinant plasmids were transferred into *E. coli* BL21 (DE3) for expression. The single clone was picked and inoculated in 3 ml of LB liquid medium containing 100 g/mL of ampicillin, and then placed in a small amount of the amplification culture at 37°C and 180 rpm/min in a shaker for 6~8 hours. From the small amount of amplification culture, 1 ml of the bacterium solution was aspirated in 300 ml of LB liquid medium containing 100g/mL ampicillin and then placed in a large amount of amplification culture at 37°C, 180 rpm/min in a shaker. When the optical density OD value of the bulk culture reached 0.6, isopropyl β- d -thiogalactoside (IPTG) inducer at a final concentration of 1 mmol/L was added to the bacterial broth to induce the protein expression. The cultures were then centrifuged at 4000 rpm for 10 min at 4°C, and the supernatant was discarded to collect the bacterial precipitate, to which lysate (10 mM Tris-HCl [pH = 8.0], 0.5% Triton X-100) was added for resuspension (lysate to bacterial solution ratio of 1:30). The lysates were lysed by ultrasonication in an ice bath (15s working, 20s interval, 10min total), and the supernatants and precipitates were subjected to sodium dodecyl sulfate-polyacrylamide gel (SDS-PAGE) electrophoresis, respectively, to identify the expression levels and forms of ESAT-6, CFP-10, nPstS1 and ECP001f. Inclusion body expressed proteins were denatured with 8M urea. Finally, the four proteins were purified from the inclusion bodies or supernatants by nickel column affinity chromatography. The inclusion body proteins were denatured with 20 mM Tris-HCl (pH = 8.0), the proteins were concentrated by ultrafiltration tubes, the protein concentrations were determined by BCA protein assay kit, and finally, the proteins were sterilely filtered through 0.22 μm filters and dispensed, and stored frozen at -80°C.

### Preparation of vaccines

The total amount of 50 μg mixture antigen ECP001m was prepared by mixture ESAT-6, CFP-10 and nPstS1 in equimolar proportions, PBS was added to the ECP001m so that the total mass of the ECP001m was 500 μg/1.5 ml, 500 μg of ECP001m contained 149.75 μg, 152.43 μg and 197.82 μg of ESAT-6, CFP-10 and nPstS1, respectively. 500 μL of aluminum adjuvant was then added to the ECP001m to prepare the vaccine. PBS was added to the fusion antigen ECP001f to give a total mass of 500 μg/1.5 ml and then 500 μl of aluminum adjuvant was added to the ECP001f to prepare the vaccine.

### Grouping of mice and immunization with vaccines

30 mice were randomly divided into 5 groups, with 6 mice in each group. Including a negative control group: PBS group; a blank control group: adjuvant group; two experimental groups: ECP001f-adjuvant group and ECP001m-adjuvant group and a positive control group: BCG vaccine group. Before the first immunization, the background serum was collected from the fundus vein of mice. In the BCG group, only 1 × 10^6^ CFU BCG vaccine was injected subcutaneously on the back at the 0th week, and the other four groups were immunized subcutaneously on the back once at the 0th, 2nd and 4th week respectively for 3 times. Each mouse in the PBS group was immunized with 200 μl PBS, each mouse in the adjuvant group was immunized with 200 μl adjuvant, each mouse in the ECP001f-adjuvant group was immunized with 200 μl ECP001f-adjuvant, and each mouse in the ECP001m-adjuvant group was immunized with 200 μl ECP001m-adjuvant. Two weeks after the last immunization, blood samples were collected from the fundus vein, and the mice were killed under anesthesia, followed by aseptic isolation of spleen cells immediately. Immunogenicity and protection are then tested and evaluated.

### Serum collection

Blood samples were collected from the eyes of mice, and the blood was placed at room temperature for 2 hours, followed by centrifugation at 4°C and 4000rpm for 10 minutes for serum separation, and the supernatant serum was collected and stored at -20°C.

### Determination of specific immunoglobulin G in mouse serum

Serum specific IgG, IgG1 and IgG2a antibodies were measured by enzyme-linked immunosorbent assay (ELISA). ECP001f, ECP001m and BCG whole bacterial lysate were diluted to 2 μg/mL using a coated buffer (50mM carbonate buffer, pH=9.0) with 100μL per well coated with 96 well ELISA plates (3 replicates per protein) and incubated overnight at 4°C. The plates were washed 5 times with 0.01M PBST (PBS solution contains 0.05% Tween-20,pH=7.2), then 100 μL of sealer containing 3% BSA was added to each well and incubated at 37°C for 2 h. PBST was used to wash the plate 5 times, and the serum of each group was diluted with PBS for many times, the dilution ratio was 2^6^-2^32^, the diluted serum was added into each well, and incubated at 37°C for 1h. The plates were washed with PBST for 5 times, and the sheep anti-mouse IgG, IgG1 and IgG2a antibodies labeled with horseradish peroxidase (HRP) were diluted with PBS at 1:5000. 100μL of the diluted antibody was added into each well and incubated at 37°C for 1 h. The plates were washed 5 times with PBST, then 100μL of chromo-developing substrate TMB was added to each well and incubated at 37°C for 15 min.

The reaction was terminated by adding 100 μL termination solution (2 M concentrated sulfuric acid) to each well. Finally, the absorbance at 450 nm was measured by a general microplate spectrophotometer.

### Isolation of mouse spleen cells

Mice were culled and immersed in 75% alcohol for 5 min, from which the spleen was isolated in a sterile way. A 200 mesh cell sieve was placed into the wells of a 6-well cell plate, 4 ml of lymphocyte isolate liquid was added to each well, and the mouse spleen was gently ground using the rubber end of a 5 ml syringe, and the filtrate of the ground spleen cells was collected into a 15 ml centrifuge tube. The 1 ml 1640 cell culture medium was slowly added along the side wall of the 15 ml centrifuge tube. After gradient centrifugation, the intermediate cloud-like lymphocytes were collected in the 15 ml centrifuge tube and washed with 10 ml 1640. The supernatant was re-suspended with a complete 1640 culture medium containing 10% fetal bovine serum and 1% penicillin and streptomycin double antibiotics. The cell concentration was determined by a cell counter, and then the splenocyte concentration was adjusted to 2×10^6^/ml with complete 1640 medium dilution for use in subsequent experiments.

### Enzyme-linked immunospot assay for IFN-γ and IL-4

The 96-well plate was activated by adding 1640 complete medium (200 μL/well) containing serum and then discarded after standing for 5 min at room temperature. Add 100 μL of splenocytes per well and stimulated with 2 μg corresponding antigens or BCG whole cell lysate, (each well was set up with replicate wells and 3 biological replicates were tested). Set up a negative control and a positive control. Negative control well: add 10 μL PBS stimulation; positive control well: add 500 ng ConA stimulation, covered the cell plate and placed it in a 37°C, 5% CO_2_ cell incubator overnight. ELISpot plate was removed, and discarded, 200 μL of deionized water was added to each well, and the cells were lysed by hypotonic lysis at 4°C for 10 min in the refrigerator. After discarding, the plate was washed 5 times with 200 μL washing solution, 100 μL biotin-labeled antibody was added to each well of the plate and incubated at 37°C for 1 h. After discarding, the plate was washed 5 times, 100 μL enzyme-linked affinity factor was added to the wells and incubated at 37°C for 1h. After discarding, the plate was washed 5 times and patted dry thoroughly. 100 μL/well of AEC color development solution was added to each well and color development was carried out at room temperature for 30 min with deionized water, washing the plate with deionized water to stop the substrate reaction. The ELISpot plates were dried and the SFCs (spot-forming cells) of each well was detected using an ELISpot plate reader.

### Lumminex assay for extracellular cytokines

100 μL of splenocytes were added to each well of a 96-well cell culture plate, and 10 μg of the corresponding immunoprotein was added to each well (each well was set up in duplicate and 3 biological replicates were tested), incubated at 37°C in a 5% CO_2_ incubator for 20 hours, after which the liquid and cells in the wells were collected. The expression levels of nine cytokines, including IL-2, IFN-γ, TNF-α, IL-12, GM-CSF, IL-17, IL-4, IL-6 and IL-10, were measured by the Luminex multifactor assay according to the manufacturer’s instructions using the Mouse Multiplex Cytokine Assay Kit.

### Mycobacterium tuberculosis *in vitro* growth inhibition assay

The *in vitro* growth inhibition test (MGIA) of Mycobacterium tuberculosis was performed in the Biosafety Level 3 laboratory. A few weeks before the experiment, we scraped an appropriate amount of fresh H37Rv colonies growing on Roche’s medium with an inoculation ring and placed them in the ultrasonic dispersion tube, and added 1 mL PBS into the tube. After the bacteria were dispersed with an ultrasonic dispersion device, the bacteria were placed for 10 minutes, and the OD600 value of the bacterial solution was measured, and the OD600 value was adjusted to 1. Dilute the bacterial solution 10 times with the dilution concentration of 10^-1^, 10^-2^, 10^-3^, 10^-4^, 10^-5^ and 10^-6^ respectively. Evenly coat the bacterial solution with different dilution concentrations on the 7H10 plate, and coat each plate with 50 μL. Repeat twice for each gradient. After two weeks, obtain the bacterial count (CFU) of each dilution concentration, and calculate the number of corresponding colonies with OD600 = 1. On the day of the experiment, scrape fresh H37Rv from Roche culture medium, wash with PBS and adjust the concentration of H37Rv bacterial solution to make its OD600 value 1. After gradient dilution, coat 50 CFU mycobacteria on the 7H10 plate as a blank control. After diluting H37Rv bacterial solution with PBS gradient, then the H37Rv was diluted to 100 CFU/ml using complete 1640 cell medium with resuspended cells (contains 0.05% Tween 80). 500 μL of 100 CFU/ml H37Rv and 500 μL of 2×10^6^ cells/ml splenocytes were added to a 24-well cell plate, and the splenocyte-H37Rv mixture was incubated at 37°C with 5% CO2 for 4 days. After 4 days, the mixture was mixed by pipetting with a pipette tip, then transferred to a 1.5 mL centrifuge tube and centrifuged at 12000 rpm for 10 min to collect the precipitate, and the supernatant was discarded. 500 μL of sterile tissue culture grade water was added to each well of the 24-well plate and incubated for 5 min at room temperature, then pipetted into the above precipitate, mixed by pipetting and diluted 10 times, and 50 μL of the original solution and diluted solution were taken and coated with 7H10 solid medium, respectively. 7H10 solid medium (containing 10% OADC nutrient solution) was coated, and one replicate well was set up for each group, and three biological replicates were tested. Incubate at 37°C for three weeks. Bacterial counts were performed and experimental data were expressed as log10 CFU per well sample.

### Statistical analysis

The experimental data were presented as mean ± standard deviation (SD) and were processed and plotted in statistical bar graphs using GraphPad Prism 8.0 software. Comparisons between two groups were made by t-test, and data from more than two groups were compared by one-way analysis of variance (ANOVA) and Tukey’s multiple comparison test. *P* < 0.05 was considered a statistically significant difference.

## Results

### Construction, expression and purification of recombinant proteins

**Figure 3:**
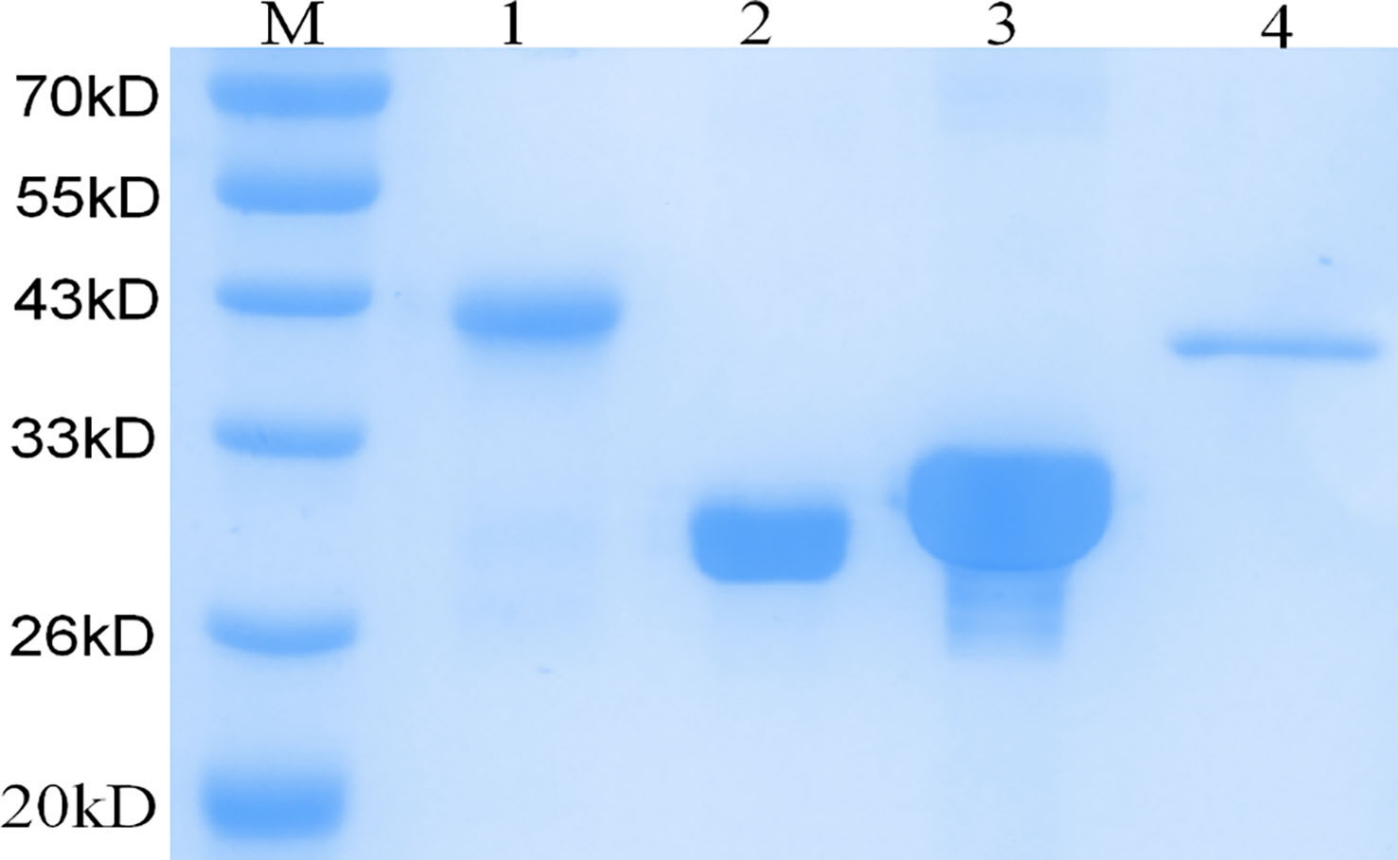
SDS-PAGE analysis of the purification and expression of recombinant protein ECP001f, ESAT6, CFP10 and nPstS1. Lane M:Prestained protein marker; Lanes 1: purified recombinant fusion protein ECP001f; Lanes 2: purified recombinant protein ESAT6; Lanes 3: purified recombinant protein CFP10; Lanes 4: purified recombinant protein nPstS1.

As confirmed by PCR and DNA sequencing, *Rv3875*, *Rv3874* and *nRv0934* were successfully cloned into the pET32a vector and the DNA sequence of ECP001f was successfully cloned into the pET43.1a vector, respectively. As shown in **Figure 3**, SDS- PAGE analysis showed that ESAT-6 and CFP-10 were expressed as supernatant proteins, nPstS1 and ECP001f were expressed as inclusion body proteins, and the molecular weights of ESAT-6, CFP-10, nPstS1 and ECP001fwere about 30.85kDa, 31.4kDa, 40.75kDa and 41.5 kDa which were consistent with the predicted results, respectively.

### Antigen-specific humoral immune response

**Figure 4:**
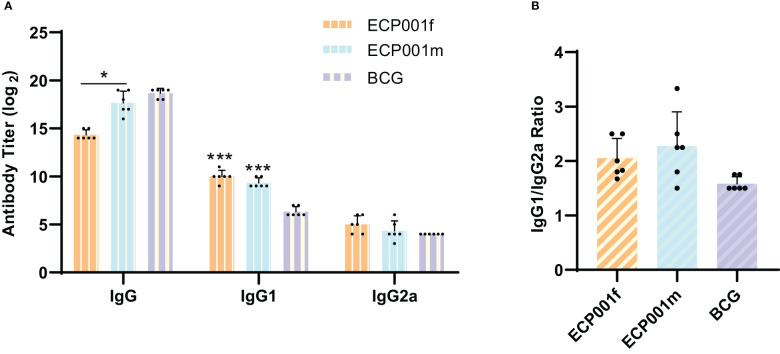
Determination of IgG, IgG1 and IgG2a subtypes in serum of BALB/C mice immunized with ECP001f, ECP001m and BCG groups. **(A)** the antibody titers of ECP001f and ECP001m groups were compared with those of BCG group. **(B)** the ratio of IgG1 to IgG2a in ECP001f and ECP001m groups was compared with that in BCG group. The significant difference between ECP001f, ECP001m group and BCG group was indicated by the asterisk above the antigen column, and the significant difference between ECP001f group and ECP001m group was indicated by the asterisk above the line between the two groups. (*P<0.05, ***P<0.001).

We selected the BCG group as the control group to evaluate the level of specific humoral immune response induced by ECP001 subunit vaccine in mice. As shown in **Figure 4A**, both ECP001f and ECP001m groups could stimulate mice to produce high titers of IgG, IgG1 and IgG2a antibodies, in which the total IgG level produced by ECP001m and BCG was higher than that of ECP001f (*P* < 0.05 and *P* < 0.01), and the IgG1 production of ECP001f and ECP001m group was higher than that of BCG group (*P* < 0.001). There was no significant difference in IgG2a level among ECP001f, ECP001m and BCG groups. There was no significant difference in the ratio of IgG1 to IgG2a induced by ECP001f, ECP001m and BCG, which was 2.06, 2.36 and 1.58 respectively (**Figure 4B**).

### ELISPOT detection of IFN-γ and IL-4 secreted cell numbers

**Figure 5:**
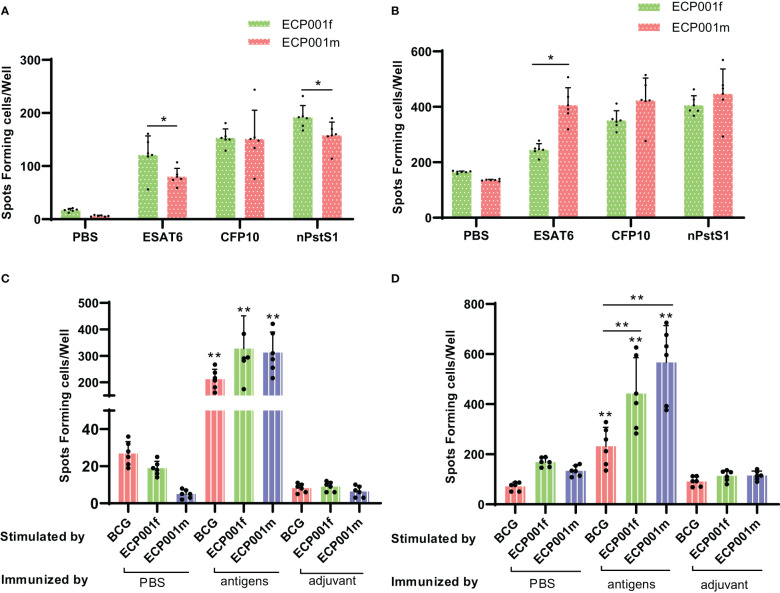
The number of IFN- γ and IL-4 secreted by mouse spleen cells stimulated by antigen or BCG whole cell lysate. The number of IFN- γ **(A)** and IL-4 **(B)** secreted by mouse spleen cells stimulated by three kinds of single antigens, and the number of IFN- γ **(C)** and IL-4 **(D)** secreted by mouse spleen cells stimulated by ECP001f, ECP001m and BCG whole cell lysate. The significant statistical difference between the vaccine or BCG whole cell lysate group and the PBS/adjuvant control group was indicated by the asterisk above the antigen group, and the significant statistical difference between the ECP001f, ECP001m group and the BCG group was indicated by the asterisk above the line between the two groups. (*P <0.05, **P<0.01).

First, antigen-specific IFN-γ and IL-4 were measured 2 weeks after the last immunization. The results showed that all three single antigens could induce the proliferation of IFN-γ and IL-4 secreting cells in ECP001f and ECP001m immunized groups (**Figures 5A, B**). The amount of IFN-γ and IL-4 secreted by mouse spleen cells stimulated by ECP001f, ECP001m and whole cell lysate of BCG was significantly higher than that of PBS and adjuvant groups (all *P*<0.01). The ability of ECP001f to stimulate mouse spleen cells to secrete IFN-γ was the strongest, but there was no significant difference among the three groups compared with ECP001m and BCG groups. The ability of ECP001m and ECP001f to stimulate the secretion of IL-4 from mouse spleen cells was significantly higher than that of the BCG group (*P*<0.01 and *P*<0.05). Among them, the ability of ECP001m to stimulate spleen cells to secrete IL-4 was the strongest, but there was no significant difference between ECP001m and ECP001f group (**Figures 5C, D**).

### Secretion of cytokines in different immune groups

**Figure 6:**
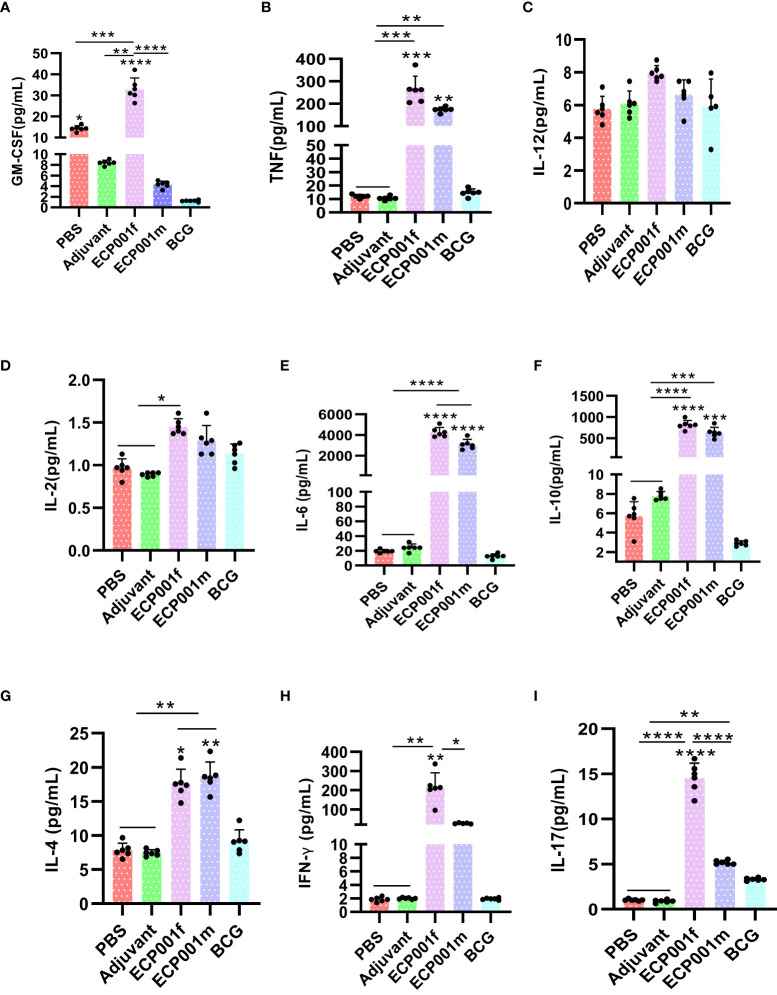
Specific cytokines produced by mouse splenocytes after *in vitro* antigen stimulation after immunization with ECP001f and ECP001m compared to PBS, Adjuvant and BCG group. **(A)** GM-CSF concentration produced by mouse splenocytes following *in vitro* antigen stimulation; **(B)** TNF-α; **(C)** IL-12; **(D)** IL-2; **(E)** IL-6; **(F)** IL-10; **(G)** IL-4; **(H)** IFN-γ; **(I)** IL-17. Statistical differences among the four groups of PBS, adjuvant, ECP001f and ECP001m were indicated by asterisks above the lines between the groups, while statistical differences between the four groups and BCG group were directly indicated by asterisks above their respective columns (*P<0.05, **P<0.01, ***P<0.001, ****P<0.0001; ns means no statistically significant).

Lumminex was used to detect the expression of nine cytokines in the spleen lymphocytes of mice from different immunization groups after stimulation with the corresponding immune proteins. As shown in **Figure 6**, first, except for IL-12, the ECP001f group secreted 8 other cytokines higher than the PBS and adjuvant groups (all *P* values < 0.05), except for IL-12 and IL-2, the ECP001f group secreted 7 other cytokines higher than the BCG group (all *P* values < 0.05), and the ECP001f group secreted GM-CSF, INF-γ and IL-17 in the ECP001f group were higher than those in the ECP001m group (*P <*0.0001, *P <*0.05 and *P <*0.0001). And then, we found except for GM-CSF, IL-12, IL-2 and INF-γ, all other cytokines were secreted higher in the ECP001m group than in the PBS and adjuvant groups (all *P* values < 0.05), and TNF, IL-6, IL-10 and IL-4 were secreted higher in the ECP001m group than in the BCG group (*P* < 0.01, *P <*0.0001, *P*< 0.001 and *P*< 0.01). At last, no statistically significant differences were found between the PBS, adjuvant and BCG groups (*P* > 0.05), except for GM-CSF secreted in the PBS group which was higher than that in the BCG group (*P* < 0.05).

### Protective efficacy of the novel subunit protein vaccine

**Figure 7:**
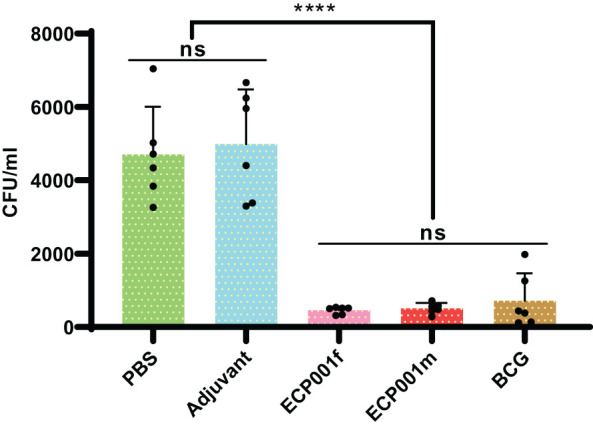
Results of the ECP001f and ECP001m groups compared with the PBS, adjuvant and BCG groups in terms of colony count. The PBS group is the negative control group; the adjuvant group is the blank control group, and the BCG group is the positive control group. Significant differences between the groups are indicated by an asterisk above the line. (ns ****P<0.0001).

Splenocytes from different immune groups were co-cultured with H37Rv for 4 days, then the cells were lysed with water, the intracellular Mycobacteria were released and smeared on the plate, the colony number of *M. tuberculosis* was calculated, and the difference of colony number among different groups was compared. The number of CFU in ECP001m, ECP001f, BCG group was significantly higher than that in PBS group and adjuvant group (all *P* values < 0.0001) respectively. There was no significant difference between PBS and adjuvant group (*P* > 0.05), and there was no significant difference in CFU number between ECP001m group, ECP001f group and BCG group (all *P* values > 0.05) (**Figure 7**).

## Discussion

The only currently available anti-tuberculosis vaccine, BCG, is virtually ineffective in preventing adult TB, its efficacy in protecting children against TB decreases over time, besides, as a live attenuated vaccine, its possible safety concerns in immunodeficient individuals. Therefore, the development of an effective and efficient TB vaccine has become a global health priority for the effective control and elimination of TB. It has been noted that BCG vaccine induces mainly effector memory T cells (TEM), which have much shorter survival time than central memory T cells (TCM) (34), which may underlie the limited duration of protection of the BCG vaccine (35). The subunit vaccine composed of recombinant protein with adjuvant can induce the body to produce more TCMs, which can rapidly differentiate into effector T cells when stimulated again by the corresponding antigen and participate in the re-response, thus leading to a long-term immune memory response (36, 37). Due to polymorphisms in the human leukocyte antigen (HLA) system, *M. tuberculosis* antigens are not recognized by the immune cells of the patients with TB. Vaccines that combine more than one antigen have the advantage of being recognized by a broad population. In addition, a vaccine based on only one antigen may encounter Mtb that does not express that antigen, so that resulting in immune evasion. Multi-component subunit protein vaccines may facilitate vaccination of genetically diverse populations and overcome antigen-presentation evasion (38, 39). Thus, recombinant protein subunit vaccines consisting of multiple *M. tuberculosis* dominant antigens with adjuvants have the advantage of being safe, providing good protection and generating long-term immune memory to provide long-lasting protection and are most likely to be a promising vaccination option.

In this study, we constructed one multi-component antigen combination, named ECP001, which includes two types, one is a mixed protein antigen named ECP001m, and the other is a fusion expression protein antigen named ECP001f, as candidates for protein subunit vaccines. The mixed antigen ECP001m and the fusion expression antigen ECP001f consisted of three single antigens, ESAT6, CFP10 and npstS1. The two antigens, ESAT6 and CFP10, are both early secreted proteins and RD region deletion antigens of *M. tuberculosis*, and the T-cell epitope coverage of the two proteins was predicted to be 100% in our laboratory. With their good immunoreactivity and ability to induce protective immunity, TB vaccines and diagnostic reagents designed on the basis of these two proteins are a hot topic and a major direction in TB research (40). However, both TB vaccines and diagnostic reagents based on these two antigens have failed to break through the research bottleneck and have not provided sufficient protection against TB and satisfactory diagnostic efficacy in humans. In the present study, we innovatively added npstS1, a tuberculosis T-cell epitope protein constructed in our laboratory, to these two proteins to construct one multi-component antigen by mixing or fusing the three proteins in order to improve the immunoreactivity of the antigen combination. As a secreted lipoprotein with good immunogenicity, PstS1 is a high-affinity phosphate-specific transporter protein that is also associated with the virulence of *M. tuberculosis* (41) and is lowly expressed in *M. tuberculosis*. PstS1 has good diagnostic efficacy (42) and has been widely used for tuberculosis diagnosis, and the serological diagnostic potential of PstS1 has been confirmed in our laboratory (43). In addition, PstS1 can increase the cellular immune response of the body represented by the Th1 type as well as induce IL-17 response (41), and it is also considered as a potential immunomodulator in tuberculosis vaccine studies. In our laboratory, we constructed nPstS1, a T-cell epitope protein, based on the predicted T-cell epitope of PstS1 combined with the immunogenicity assessment of the epitope peptide and the distribution of epitopes. NpstS1 was experimentally validated to have not only good immunoreactivity but also more immunodominant compared to PstS1, making it suitable as a TB vaccine candidate antigen (29). We prepared one novel protein subunit vaccine which named ECP001 by combining the multi-component antigen combinations with aluminum adjuvants and have demonstrated the good immunogenicity and protective properties of our vaccine by immunizing mice and observing relevant experimental indices.

### Analysis of the cellular immune effects

After *M. tuberculosis* spread to the lymph nodes, they stimulate antigen-specific T cells to proliferate and migrate to the foci of infection, forming part of the granuloma along with helper T cells such as Thl, Th2, Thl7, and regulatory T cells. These cells exert their functions mainly through the release of soluble cytokines. In general, IFN-γ producing Th1 cells play an important role in the prevention of TB by enhancing the ability of macrophages to phagocytose to kill *M. tuberculosis* (44). In addition to IFN-γ, other cytokines are involved in protective immunity against tuberculosis and play a role in bacterial control following *M. tuberculosis* infection. Granulocyte-macrophage T cells secrete cell colony-stimulating factor (GM-CSF), a hematopoietic growth factor that promotes the generation, proliferation and maturation of dendritic cells (45, 46). Mature dendritic cells can secrete a variety of cytokines with proven protective effects, such as IL-12, TNF-α and IFN-γ. These cytokines act on natural CD4+ T cells (Th0 cells) to differentiate them into Th1 cells and, in addition, mature dendritic cells produce IL-4 to differentiate Th0 cells into Th2 cells. Studies have shown that Th1-type cytokines, including IFN-γ, TNF-α and IL-2, play an important role in the control of *M. tuberculosis* infection (25, 47). IFN-γ enhances microbial clearance by macrophages mainly through activation of the inducible nitric oxide synthase pathway (48) and induction of phagosomal acidification, maturation and autophagy mechanisms (49). TNF plays a crucial role in controlling and containing intracellular pathogens, recruiting inflammatory cells to foci of infection, and stimulating granuloma formation and maintenance (50). IL-2 is an initial factor in the cellular immune response process and has a variety of promoting effects on NK or LAK cells, inhibiting Th2 cell development, selectively enhancing Th1 cell differentiation and proliferation, inducing IFN-γ secretion, generating a specific immune response, and inducing a shift from Th2 to Th1 cellular responses (51). Another major Th1-type cytokine is interleukin 12 (IL-12), through which Th1 immune induction and maintenance are enhanced. Th2 cells mediate humoral immune responses by secreting IL-4, IL-5, and IL-10, thereby affecting the function of B lymphocytes (52), and in addition, excessive Thl-type immune responses can cause pathological tissue damage. An appropriate Th2 response inhibits the overreaction of Th1 and protects tissues from damage, but an overpowering Th2 immune response, in turn, antagonizes the Th1-type cellular response that plays a major role in *M. tuberculosis* infection, leading to mycobacterial survival, thus suggesting that Th1 and Th2 responses and the appropriate balance between them appear to be closely related to optimal protective immunity (53). Th17 cells and the cytokine IL-17 can be involved in the regulatory process of Th1/Th2 cytokine function and have an important role in autoimmune diseases and resistance to intracellular bacterial infections. Studies have shown that Th17 cells are involved in regulating the immune response to tuberculosis and their secreted IL-17 is involved in anti-infective immunity at both the level of intrinsic and adaptive immunity (54).

In this study, a total of nine protective cytokines, antigen-specific IL-2, IFN-γ, TNF-α, IL-4, IL-6, IL-10, IL-12, GM-CSF and IL-17, were detected using ELISPOT and Luminex techniques. The results showed that the levels of 8 cytokines secreted by splenic lymphocytes in ECP001f and ECP001m groups were all higher than those in PBS group, adjuvant group and BCG group, except GM-CSF. Among them, the level of IFN-γ, which represents the Th1 type immune response, secreted by the ECP001f group was higher than that of the ECP001m group, while the level of IL-4, which represents the Th2 type immune response, secreted by the ECP001m group was higher than that of the ECP001f group. In addition, the levels of several protective cytokines secreted in the ECP001f group were also higher than those in the ECP001m group, except for IL-4, indicating that the ECP001f group had the strongest ability to induce the body to produce various protective cytokines in addition to the strongest ability to produce Th1-type cellular immune responses.

### Analysis of the humoral immune effects

IgG2a usually represents Th1-type cellular immunity, which mediates mainly cellular immune responses, while IgG1 usually represents Th2-type cellular immunity, which mediates mainly humoral immune responses, and the type of immune response was determined by calculating the ratio of IgG1 to IgG2a. Our results showed that the ratio of IgG/IgG2a was greater than 1 in both the ECP001f and ECP001m groups, indicating that both induced a BCG-similar immune response favoring the Th2 type, suggesting that the two multi-component protein antigens mixed with aluminum adjuvant have a strong ability to induce humoral immunity. This may be related to the use of aluminum adjuvants, which are usually predominant in promoting antibody-mediated protective immune responses (55, 56). Although cell-mediated immunity has been shown to play a critical role in protection against and clearance of *M. tuberculosis* infection, humoral immunity can also enhance immune protection against *M. tuberculosis* infection in humans in different ways: There is evidence that intradermal BCG vaccination induces IgG and IgM secretion to recognize several *Mycobacterium* antigens (57–59). Some of these antibodies can be based on multiple mechanisms to enhance cellular and humoral immunity against *M. tuberculosis* infection, including accelerating phagocytic fusion, promoting clearance of immunomodulatory antigens, and influencing the outcome of mycobacterial infection through the ability to modulate inflammation (57). In addition, antibodies modulate immunity through Fc receptor-mediated phagocytosis, thus providing protection against intracellular pathogens (59).

As illustrated by the above experimental results, the fusion protein ECP001f can produce both a significant Th1-type response and a certain degree of Th2-type response. In addition, by testing cytokines in the population vaccinated with clinical phase subunit vaccines H1:IC31, H56:IC31, M72/AS01E, and ID93+GLA-SE, it was found that none of them produced IL-17, indicating that all of them failed to successfully induce a Th17-type immunoprotective response, while our results showed that the level of IL-17 in the ECP001f group was significant, which suggests that ECP001f seems to induce a Th1-Th2 immune homeostatic response and a Th17-type immunoprotective response.

### Analysis of anti-mycobacterium infection effects

At present, the protective evaluation of the vaccine mainly depends on challenge *in vivo* to observe the ability to inhibit the replication of *Mycobacteria* after immunization. Some studies have found that mycobacterium growth inhibition assay *in vitro* (MGIA) can connect *in vitro* functional test with protective effect. Compared with unimmunized mice, there is repeatable mycobacterial growth inhibition in spleen cells of mice immunized with BCG, and the inhibitory effect is consistent with the *in vivo* bacteriostatic effect of post-immunization challenge test (60). Therefore, the evaluation of vaccine effectiveness with MGIA is considered as a screening strategy for selecting candidate vaccines or quickly entering expensive clinical development paths (60). Using MGIA to observe the effect of the vaccine on the proliferation of *M. tuberculosis*, although there is a certain gap with the evaluation of immune-animal challenge-bacterial load, it can also provide a certain basis for the prediction of immune protection performance of the vaccine. The MGIA results of this study showed that the CFU of spleen lymphocytes co-cultured with H37Rv in ECP001m and ECP001f groups was significantly lower than that in PBS and adjuvant groups, indicating that both of them could inhibit the growth of *M. tuberculosis in vitro*, and there was no significant difference between CFU in ECP001m and ECP001f group and BCG group, indicating that the inhibitory effect *in vitro* was similar to that of BCG, suggesting that both of them could induce mice to produce immune protection against *M. tuberculosis* infection.

The above experimental results are described with BCG group as the control group. In the follow-up study, we will take ESAT6 and CFP10 as the control group for further evaluation. In short, one novel multi-component protein antigen was developed in this study, named ECP001, which includes two types. The fusion expression protein ECP001f constructed by genetic engineering of single dominant antigen ESAT6, CFP10 and nPstS1 and the mixed protein ECP001m formed by the mixture of three antigens in equal proportion, which combined with aluminum adjuvant to form one novel multicomponent subunit protein vaccine. The vaccine can induce strong humoral and cellular immune responses in mice, which can significantly inhibit the growth of *M. tuberculosis* and provide good protective effect.

The multi-component vaccine ECP001 we studied contains two single antigens, they are ESAT6 and CFP10. Because they are the early dominant antigens secreted by Mtb, they have good diagnostic value, and they are included in many methods for the diagnosis of tuberculosis infection, such as Skin tests (including Diaskintest,C-Tbskintest and EC-Test) and IGRAs (including T-SPOT.TB, QFT-GIT, QFT-plus, LIAISONQFT-Plus and LIOFeron TB/LTBI), if the ECP001 we study in the future has the opportunity to be a tuberculosis vaccine. It is inevitable that there will be interference with these above diagnostic methods. However, because ESAT-6 and CFP-10 are also antigens with strong immunogenicity and immune protection, they have good application value in the development of tuberculosis vaccine. For example, H56/IC31 which has entered the clinical IIb phase also contains ESAT6, H56/IC31 as a promising protein subunit vaccine is still being studied. Our results also confirm the good immune effect and potential vaccine application value of ESAT-6 and CFP-10. Secondly, there are other diagnostic methods that are more sensitive and specific than immunological methods in the diagnosis of tuberculosis. For example, phage detection MtbDNA (61) or digital PCR detect whole blood MtbDNA (62) et al.

It can be concluded that ECP001 is a novel effective multicomponent subunit vaccine candidate with potential as prophylactic or therapeutic vaccine for *M. tuberculosis* infection. In subsequent studies, we will try to change the vaccination route or select more suitable animal models and delivery systems for further exploration. In addition, the fusion protein ECP001f could be combined with latency-associated antigens to construct a multistage subunit vaccine or expressed in BCG to construct a live vaccine to determine its full potential.

## Data availability statement

The data presented in the study are deposited in the NCBI GenBank repository, accession numbers OQ709971, OQ709972, OQ709973, OQ709974.

## Ethics statement

The animal study was reviewed and approved by the Ethics Committee of the National Institute for Communicable Disease Control and Prevention, Chinese Center for Disease Control and Prevention.

## Author contributions

JY, XF, and KW designed the study. HL contributed to the epitope prediction. JY, XL, RW, BC and CQ performed experiments. JY and GL analyzed and interpreted data. KW provided reagents. JY and KW wrote the manuscript. All authors contributed to the article and approved the submitted version.
