# Supplementary material for: A novel multi-component protein vaccine ECP001 containing a protein polypeptide antigen nPstS1 riching in T-cell epitopes showed good immunogenicity and protection in mice
Source: Front Immunol. 2023 Apr 21;14:1138818. doi: 10.3389/fimmu.2023.1138818 (PMC10161251; doi:10.3389/fimmu.2023.1138818)
Supplement: Supplementary file 1 [file DataSheet_1.pdf]

Nucleotide sequence of ECP001f

ATGACAGAGCAGCAGTGGAATTTCGCGGGTATCGAGGCCGCGGCAAGCGC

AATCCAGGGAAATGTCACGTCCATTCATTCCCTCCTTGACGAGGGGAAGCA

GTCCCTGACCAAGCTCGCAGCGGCCTGGGGCGGTAGCGGTTCGGAGGCGT

ACCAGGGTGTCCAGCAAAAATGGGACGCCACGGCTACCGAGCTGAACAAC

GCGCTGCAGAACCTGGCGCGGACGATCAGCGAAGCCGGTCAGGCAATGGC

TTCGACCGAAGGCAACGTCACTGGGATGTTCGCAGGTGGTTCTGGCGGTG

CAGAGATGAAGACCGATGCCGCTACCCTCGCGCAGGAGGCAGGTAATTTC

GAGCGGATCTCCGGCGACCTGAAAACCCAGATCGACCAGGTGGAGTCGAC

GGCAGGTTCGTTGCAGGGCCAGTGGCGCGGCGCGGCGGGGACGGCCGCC

CAGGCCGCGGTGGTGCGCTTCCAAGAAGCAGCCAATAAGCAGAAGCAGG

AACTCGACGAGATCTCGACGAATATTCGTCAGGCCGGCGTCCAATACTCGA

GGGCCGACGAGGAGCAGCAGCAGGCGCTGTCCTCGCAAATGGGCTTCGGT

GGTTCTGGCGGTGGTAGCACGCTGCTCTACCCGCTGTTCAACCTGTGGGGT

CCGGCCTTTCACGAGAGGTATCCGAACGTCACGATCACCGCTCAGGGCACC

GGTTCTGGTGCCGGGATCGCGCAGGCCGCCGCCGGGACGGTCAACATTGG

GGCCTCCGACGCCTATCTGTCGGAAGGTGATATGGCCGCGCACAAGGGGCT

GATGAACATCGCGCTAGCCATCTCCGCTCAGCAGGTCAACTACAACAATAG

CTCTGGCAATTTCTTGTTGCCCGACGCGCAAAGCATTCAGGCCGCGGCGGC

TGGCTTCGCATCGAAAACCCCGGCGAACCAGGCGATTTCGATGATCGACGG

GCCCGCCCCGGACGGCTACCCGATCATCAACTACGAGTACGCCATCGTCAA

CAACCGGCAAAAGGACGCCGCCACCGCGCAGACCTTGCAGGCATTTCTGC

ACTGGGCGATCACCGACGGCAACAAGGCCTCGTTCCTCGACCAGGTTCATT

TCCAGCCGCTGCCGCCCGCGGTGGTGAAGTTGTCTGACGCGTTGATCGCG

ACGATTTCCTAG

Amino acid sequence of ECP001f

MTEQQWNFAGIEAAASAIQGNVTSIHSLLDEGKQSLTKLAAAWGGSGSEAYQ
GVQQKWDATATELNNALQNLARTISEAGQAMASTEGNVTGMFAGGSGGAE
MKTDAATLAQEAGNFERISGDLKTQIDQVESTAGSLQGQWRGAAGTAAQAA
VVRFQEAANKQKQELDEISTNIRQAGVQYSRADEEQQQALSSQMGFGGSGG
GSTLLYPLFNLWGPAFHERYPNVTITAQGTGSGAGIAQAAAGTVNIGASDAYL
SEGDMAAHKGLMNIALAISAQQVNYNNSSGNFLLPDAQSIQAAAAGFASKTP
ANQAISMIDGPAPDGYPIINYEYAIVNNRQKDAATAQTLQAFLHWAITDGNKA
SFLDQVHFQPLPPAVVKLSDALIATIS*

Nucleotide sequences of individual proteins in ECP001m

Rv3875

ATGACAGAGCAGCAGTGGAATTTCGCGGGTATCGAGGCCGCGG
CAAGCGCAATCCAGGGAAATGTCACGTCCATTCATTCCCTCCTT
GACGAGGGGAAGCAGTCCCTGACCAAGCTCGCAGCGGCCTGG
GGCGGTAGCGGTTCGGAGGCGTACCAGGGTGTCCAGCAAAAAT
GGGACGCCACGGCTACCGAGCTGAACAACGCGCTGCAGAACC
TGGCGCGGACGATCAGCGAAGCCGGTCAGGCAATGGCTTCGAC
CGAAGGCAACGTCACTGGGATGTTCGCA

Rv3874

ATGGCAGAGATGAAGACCGATGCCGCTACCCTCGCGCAGGAGG
CAGGTAATTTCGAGCGGATCTCCGGCGACCTGAAAACCCAGAT
CGACCAGGTGGAGTCGACGGCAGGTTCGTTGCAGGGCCAGTG

GCGCGGCGCGGCGGGGACGGCCGCCCAGGCCGCGGTGGTGCG
CTTCCAAGAAGCAGCCAATAAGCAGAAGCAGGAACTCGACGA
GATCTCGACGAATATTCGTCAGGCCGGCGTCCAATACTCGAGGG
CCGACGAGGAGCAGCAGCAGGCGCTGTCCTCGCAAATGGGCTT
C

nRv0934

GGTAGCACGCTGCTCTACCCGCTGTTCAACCTGTGGGGTCCGG
CCTTTCACGAGAGGTATCCGAACGTCACGATCACCGCTCAGGG
CACCGGTTCTGGTGCCGGGATCGCGCAGGCCGCCGCCGGGACG
GTCAACATTGGGGCCTCCGACGCCTATCTGTCGGAAGGTGATAT
GGCCGCGCACAAGGGGCTGATGAACATCGCGCTAGCCATCTCC
GCTCAGCAGGTCAACTACAACAATAGCTCTGGCAATTTCTTGTT
GCCCGACGCGCAAAGCATTCAGGCCGCGGCGGCTGGCTTCGCA
TCGAAAACCCCGGCGAACCAGGCGATTTCGATGATCGACGGGC
CCGCCCCGGACGGCTACCCGATCATCAACTACGAGTACGCCATC
GTCAACAACCGGCAAAAGGACGCCGCCACCGCGCAGACCTTG
CAGGCATTTCTGCACTGGGCGATCACCGACGGCAACAAGGCCT
CGTTCCTCGACCAGGTTCATTTCCAGCCGCTGCCGCCCGCGGTG
GTGAAGTTGTCTGACGCGTTGATCGCGACGATTTCC

Amino acid sequences of individual proteins in ECP001m

ESAT-6

MTEQQWNFAGIEAAASAIQGNVTSIHSLLDEGKQSLTKLAAAWG

GSGSEAYQGVQQKWDATATELNNALQNLARTISEAGQAMASTEG

NVTGMFA

CFP-10

AEMKTDAATLAQEAGNFERISGDLKTQIDQVESTAGSLQGQWRG

AAGTAAQAAVVRFQEAANKQKQELDEISTNIRQAGVQYSRADEE

QQQALSSQMGF

nPstS1

GSTLLYPLFNLWGPAFHERYPNVTITAQGTGSGAGIAQAAAGTVNI

GASDAYLSEGDMAAHKGLMNIALAISAQQVNYNNSSGNFLLPDA

QSIQAAAAGFASKTPANQAISMIDGPAPDGYPIINYEYAIVNNRQK

DAATAQTLQAFLHWAITDGNKASFLDQVHFQPLPPAVVKLSDALI

ATIS
